# Pre-transplant hepatic steatosis (fatty liver) is associated with chronic graft-vs-host disease but not mortality

**DOI:** 10.1371/journal.pone.0238824

**Published:** 2020-09-11

**Authors:** Ko Maung, Sendhilnathan Ramalingam, Mohammad Chaudhry, Yi Ren, Sin-Ho Jung, Kristi Romero, Kelly Corbet, Nelson J. Chao, Taewoong Choi, Anna Mae Diehl, Louis Diehl, Cristina Gasparetto, Mitchell Horwitz, Gwynn Douglas Long, Richard D. Lopez, David A. Rizzieri, Stefanie Sarantopoulos, Keith M. Sullivan, Mustafa R. Bashir, Anthony D. Sung

**Affiliations:** 1 Division of Hematologic Malignancies and Cell Therapy, Department of Medicine, Duke University School of Medicine, Durham, North Carolina, United States of America; 2 Department of Radiology, Duke University School of Medicine, Durham, North Carolina, United States of America; 3 Duke Cancer Institute, Biostatistics Shared Resources, Duke University, Durham, North Carolina, United States of America; 4 Department of Biostatistics and Bioinformatics, Duke University School of Medicine, Durham, North Carolina, United States of America; 5 Duke Cancer Institute, Duke University, Durham, North Carolina, United States of America; 6 Division of Gastroenterology, Department of Medicine, Duke University School of Medicine, Durham, North Carolina, United States of America; Texas A&M University, UNITED STATES

## Abstract

Allogeneic-HCT (allo-HCT), while potentially curative, can result in significant complications including graft versus host disease (GVHD). Prior studies suggest that metabolic syndrome may be one risk factor for GVHD. We hypothesized that hepatic steatosis on pre-HCT computed tomography (CT) scans may be a marker for development of GVHD and poor outcomes in allo-HCT. In this retrospective study, we reviewed the pre-HCT CT scans and transplant outcome data of patients who underwent allo-HCT at Duke University Medical Center from 2009 to 2017. The presence of steatosis was confirmed using CT attenuation measurements. We then assessed the association between pre-HCT hepatic steatosis and HCT-related outcomes including GVHD. 80 patients who had pre-HCT CT scans were included in the study. Pre-transplant hepatic steatosis was associated with the development of chronic GVHD (OR 4.2, p = 0.02), but was not associated with acute GVHD (OR 1.3, p = 0.7), non-relapse mortality (p = 0.81) or overall survival (p = 0.74). Based on this single center retrospective study, pre-transplant hepatic steatosis is associated with development of chronic GVHD. Further, prospective study with other imaging modalities including non-contrasted CT scans is needed to determine if this association is reproducible.

## Introduction

Allogeneic hematopoietic stem cell transplant (allo-HCT) can be a curative procedure for malignant and non-malignant hematologic conditions. Despite its benefits, allo-HCT is complicated by significant transplant-related mortality (TRM) [1–3] of which graft-vs-host disease (GvHD) is the main contributor. Several laboratory biomarkers of GVHD including inflammatory cytokines have been proposed [4–6], however none are consistently used in standard clinical practice.

Patient-specific factors may also help identify patients at risk of developing GVHD. HCT-related outcomes may be related to several patient-specific variables including underlying disease, age and performance status [7]. However, few patient-specific variables beyond age predict GvHD [8, 9].

Steatohepatitis is a condition characterized by abnormal lipid deposition in the liver with associated hepatic inflammation and fibrosis. Hepatic steatosis, the precursor to steatohepatitis, occurs in about 75% [10] of patients with metabolic syndrome/obesity, which has previously been associated with poor outcomes in allo-HCT including GVHD and TRM [11–13]. Furthermore, many of the inflammatory cytokines implicated in the development of GvHD have also been known to be involved in hepatic steatosis: Evidence suggests that induction of inflammation from adipose tissue of obese subjects contributes to obesity induced nonalcoholic fatty liver disease in which the IL-1 cytokine superfamily play a role [14] and serum IL-6 levels are higher in animal models and patients with fatty liver disease [15, 16]. Also, obesity and metabolic syndrome are known to be associated with changes in gut microbiota [17, 18] and recent studies have implicated changes in gut microbiota and inflammatory immune response within the digestive tract in transplant outcomes like GvHD [19, 20].

While pre-HCT transaminitis has previously been associated with development of acute GvHD of the liver [21], many patients with hepatic steatosis will not have laboratory-detectable elevation in their transaminases [22]. Liver biopsy is the gold standard for diagnosing hepatic steatosis, however noninvasive tests are available including magnetic resonance imaging, ultrasound and computed tomography (CT) with newer imaging technologies are increasingly available [23]. Because of the availability of prior CT scans in the allo-HCT population, we chose to focus on this imaging modality in which hepatic steatosis manifests as reduced attenuation of the liver parenchyma due to triglyceride accumulation [24, 25]. We hypothesized that pre-HCT hepatic steatosis detected by CT scan may be associated with increased risk of HCT-related complications including GvHD.

## Methods

Duke Institutional Review Board approved this study (eIRB 00093882). The data were analyzed anonymously.

### Study population

692 patients underwent 744 allogeneic stem cell transplants from 1/1/2009 to 12/31/2017. 177 patients underwent contrast-enhanced CT (73% of all CT examinations) of the abdomen and pelvis within 1 year prior to their allo-HCT. To reduce confounding factors, 77 CTs performed for acute indications were excluded. A further 20 CTs were excluded as follows: 11 patients had a prior allo-HCT (and did not have a CT before the first transplant), 2 had splenectomy, and 7 only had arterial phase CT, which would have confounded attenuation measurements. Patients with prior splenectomy were also excluded due to necessity for normalization of attenuation measurements using the spleen to detect hepatic steatosis. The remaining 80 CTs were included in the study.

### Imaging diagnosis of fatty liver disease

**Fig 1:**
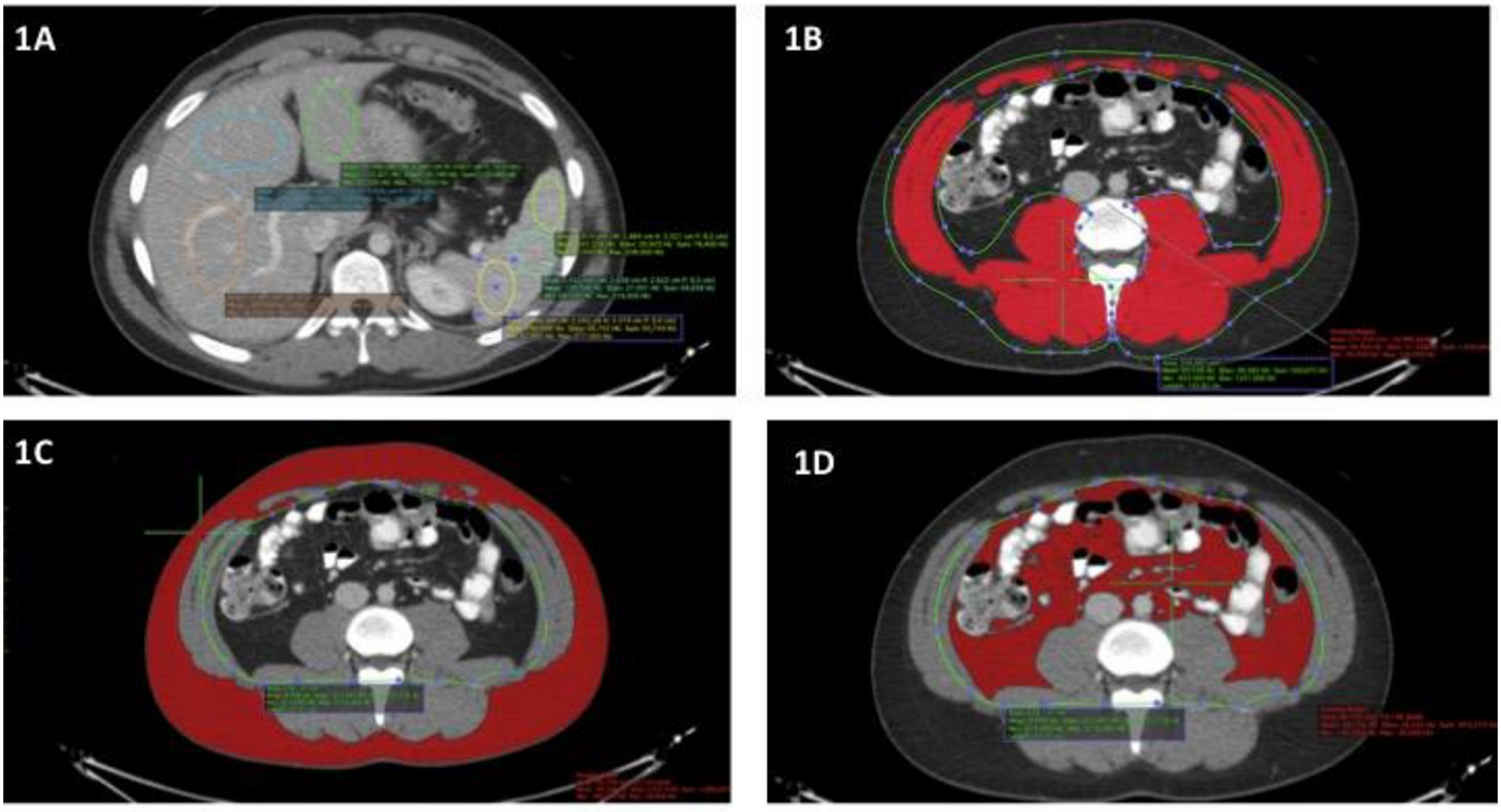
a: Liver and spleen attenuation measurements. Attenuation (in HU) of liver and spleen was measured using three regions of interest, each at least 2 cm^2^ in area to ensure measurements are representative of the organ. A difference between mean spleen and liver attenuations of at least 10 HU was used to determine presence of hepatic steatosis. b: Skeletal muscle cross-sectional area at L3 level. Skeletal muscle at the L3 level was outlined (green line). Then, an attenuation threshold between -29HU and +150HU was applied to refine the region of interest on a pixel-wise basis to determine the cross-sectional area of muscle (red). c: Subcutaneous fat measurement at L3. For subcutaneous fat, the abdominal cavity was outlined (green line), An attenuation threshold of -30HU or less was applied outside the abdominal cavity to refine the region of interest on a pixel-wise basis to determine the cross-sectional area of the subcutaneous fat (red). d: Visceral muscle -sectional area at L3. For visceral fat, the abdominal cavity was outlined (green line) and an attenuation threshold of -30HU or less was applied inside the abdominal cavity to refine the region of interest on a pixel-wise basis to determine the cross-sectional area of visceral fat (red).

We used the OsiriX image viewer (Pixmeo, Geneva, Switzerland) for image assessment. Three regions of interest, each containing an area of at least 2 cm^2^, were placed in both the liver and spleen as in Fig 1a. We used the widely accepted attenuation difference of at least 10 Hounsfield units (HU) between the mean attenuations of the spleen and liver to determine the presence of hepatic steatosis. For contrast-enhanced CTs, this has been associated with sensitivity, specificity, positive predictive value and negative value of 60.5%, 100%, 100% and 96.9% for the diagnosis of hepatic steatosis [26].

### Measurement of abdominal adiposity and skeletal muscle

We also measured the cross-sectional areas of the visceral fat, subcutaneous fat and skeletal muscle at the level of the third lumbar vertebral body level (L3) (Fig 1b, 1c and 1d). The areas of interest were traced and refined using attenuation thresholds for fat (-190 HU to -30 HU) and skeletal muscle (-29HU to +150HU). Intramuscular fat was considered to be part of the subcutaneous fat.

### Statistical considerations

Patient characteristics were summarized as count (%) for categorical variables and median (interquartile range) for continuous variables for all patients. To test for differences between patients with and without hepatic steatosis, Chi-square or Fisher’s exact test were used to compare categorical variables, and Wilcoxon Rank Sum tests or t-tests were used to compare continuous variables, as appropriate.

Because of limited sample size and event rate, number of covariates was limited by performing stepwise selection. Candidate variables that did not reach the preset criteria were excluded to avoid overfitting of the model.

Logistic regression analysis was used for prediction of acute GvHD (grade II-IV vs. 0/I) and chronic GvHD (grade I-IV vs. 0), with stepwise selection with significance of entry = 0.1 and significance of stay = 0.2. Candidate variables including race, sex, disease, conditioning regimen, and donor type, with hepatic steatosis were assessed. Time to GvHD development was also examined by non-parametric Kaplan-Meier curves with log-rank test and multivariate Cox proportional hazard regression.

Overall survival (OS) was defined as the time from diagnosis to death or last follow-up. Cox proportional hazards regression analyses were utilized to estimate the association between variables of interest and OS. Similar stepwise selection was used on the same set of variables.

Non-relapse mortality (NRM) was defined as any death in the absence of relapse, with relapse treated as a competing event. Cumulative incidence plots with Gray’s K-sample tests were used as univariate comparison of NRM in terms hepatic steatosis. A Fine-Gray sub-distribution model was used to estimate the association between multiple variables and NRM, with stepwise selection with significance of entry = 0.05 and significance of stay = 0.1. All analyses were conducted using SAS version 9.4 (SAS Institute, Cary, NC).

## Results

### Patient characteristics

Compared to the entire cohort of 692 patients, patients in the study cohort tended to have lower median age (49 years vs 52.5 years), higher likelihood of transplant diagnosis lymphoma (84% vs 13%, likely related to imaging used for lymphoma restaging prior to HCT which is not routinely done for leukemias and other diseases), higher likelihood of non-myeloablative conditioning (61% vs 36%), and higher likelihood of a peripheral blood graft (89% vs 73%); further detailed comparisons are shown in S1 Table.

Of 80 CTs, 59 (74%) had hepatic steatosis as defined by the difference in attenuation between the spleen and the liver. Patients with hepatic steatosis tended to be older, though this difference was not statistically significant (median age of 52 vs. 38 years, p = 0.052). Between those with and without hepatic steatosis, there were no statistically significant differences in gender, race, ethnicity, related donors, height, weight, Karnofsky Performance Status, HCT comorbidity index, history of diabetes, history of coronary artery disease, history of elevated liver function tests, primary disease, conditioning regimen, or stem cell source (Table 1a).

**Table 1:** a. Patient Characteristics. b. Imaging Findings and Clinical Outcomes.

A				
	All Patients	Hepatic Steatosis	No Hepatic Steatosis	
	N = 80 (100%)	N = 59 (73.7%)	N = 21 (26.2%)	P-Value
Age at Transplant (years)				
Median (IQR)	49 (34.5–57.5)	52 (39–58)	38 (29–54)	0.052
Sex				
Female	31 (38.8%)	23 (39%)	8 (38.1%)	0.94
Pre-transplant Weight (kg)				
Median (IQR)	80.15 (70.75–92.5)	78.6 (71.3–92.6)	81.8 (68–91)	0.82
Height (cm)				
Median (IQR)	172.8 (164.5–179.05)	172 (166–179)	174 (163–179.1)	0.97
Race				
White	64 (80%)	47 (79.7%)	17 (81%)	>0.99
Black	14 (17.5%)	10 (16.9%)	4 (19%)	
American Indian	1 (1.3%)	1 (1.7%)	0 (0%)	
Asian	1 (1.3%)	1 (1.7%)	0 (0%)	
Disease				
Lymphomas	54 (67.5%)	41 (69.5%)	13 (61.9%)	0.39
MDS/MPN/Other	20 (25%)	15 (25.4%)	5 (23.8%)	
Acute Leukemias	6 (7.5%)	3 (5.1%)	3 (14.3%)	
Conditioning Class				
Non-myeloablative	49 (61.3%)	39 (66.1%)	10 (47.6%)	0.19
Myeloablative	31 (38.8%)	20 (33.9%)	11 (52.4%)	
Cell Type				
Peripheral Blood Progenitor Cells	71 (88.8%)	53 (89.8%)	18 (85.7%)	0.66
Cord Blood	7 (8.8%)	5 (8.5%)	2 (9.5%)	
Bone Marrow	2 (2.5%)	1 (1.7%	1 (4.8%)	
Donor Type				
Unrelated	44 (55%)	29 (49.2%)	15 (71.4%)	0.08
Related	36 (45%)	30 (50.8%)	6 (28.6%)	
HLA Match				
Fully Matched	65 (81.3%)	47 (79.7%)	18 (85.7%)	0.75
Unmatched	15 (18.8%)	12 (20.3%)	3 (14.3%)	
Post-transplant Cyclophosphamide	16 (20%)	9 (15.3%)	7 (33.3%)	0.11
Anti-thymocyte Globulin	9 (11.3%)	6 (10.2%)	3 (14.3%)	0.69
KPS				
< = 80	43 (53.8%)	32 (54.2%)	11 (52.4%)	0.88
>80	37 (46.3%)	27 (45.8%)	10 (47.6%)	
HCT-CI				
< = 3	52 (65%)	37 (62.7%)	15 (71.4%)	0.47
>3	28 (35%)	22 (37.3%)	6 (28.6%)	
Diabetes Mellitus Type II	15 (18.8%)	13 (22%)	2 (9.5%)	0.21
Hyperlipidemia/dyslipidemia	14 (17.5%)	12 (20.3%)	2 (9.5%)	0.26
Coronary artery disease	4 (5%)	3 (5.1%)	1 (4.8%)	0.95
Alcohol Abuse	1 (1.3%)	1 (1.7%)	0 (0%)	>0.99
AST				
Median (IQR)	25.5 (19.5–35)	26 (20.5–37)	21.5 (16–31.5)	0.19
ALT				
Median (IQR)	25.5 (16.5–48.5)	26 (16–58)	23.5 (17.5–31.5)	0.47
Alkaline Phosphatase				
Median (IQR)	68.5 (54.5–85)	70 (58.5–90)	58 (44.5–71)	0.10
Bilirubin Total				
Median (IQR)	0.65 (0.5–0.8)	0.6 (0.5–0.9)	0.7 (0.5–0.8)	0.78
Albumin				
Median (IQR)	3.3 (3.1–3.8)	3.35 (3.15–3.7)	3.25 (3–3.85)	0.59
Hemoglobin A1C				
Median (IQR)	5.35 (5.15–6.5)	5.3 (5–7.6)	5.4 *	N/A
**B**				
**Imaging Findings**:				
Subcutaneous fat cross-sectional area at L3 (cm^2^)				
Median (IQR)	210.17 (148.13–318.82)	207.41 (148.19–318.26)	212.93 (128.13–319.13)	0.91
Visceral fat cross-sectional area at L3 (cm^2^)				
Median (IQR)	118.1 (77.17–207.65)	118.55 (77.91–217.82)	117.64 (74.52–171.29)	0.50
Skeletal muscle cross-sectional area at L3 level (cm^2^)				
Median (IQR)	162.9 (128.85–186.32)	158.52 (128.15–187.57)	168.3 (129.56–182.98)	0.89
**Clinical Outcomes**:				
Acute Graft vs Host Disease, grade II-IV	53 (66.3%)	39 (66.1%)	14 (66.7%)	>0.99
Chronic Graft vs Host Disease	35 (43.8%)	29 (49.2%)	6 (28.6%)	0.13
Days to discharge from Transplant				
Median (IQR)	85 (63.5–98)	85 (65–99)	83 (57–92)	0.99

*Only one patient in No Hepatic Steatosis group had HbA1C data.

MDS: myelodysplastic syndrome; MPN: myeloproliferative neoplasm; HLA: human leukocyte antigens; KPS: Karnofsky Performance Score; HCT-CI: Hematopoietic Cell Transplantation-Comorbidity Index; AST: Aspartate transaminase; ALT: Alanine transaminase

While the exact cause of steatosis was not directly ascertainable, out of 59 patients with hepatic steatosis, 43 had one or more of following diagnoses that are often associated with nonalcoholic fatty liver disease: overweight/obesity (per CDC definition of BMI > 25), hyperlipidemia, diabetes mellitus. In addition, one patient had a history of alcohol abuse.

### Acute GvHD, grade II-IV

Acute GVHD occurred in 39 patients (66.1%) in the hepatic steatosis group and 14 patients (66.7%) among those without pre-HCT hepatic steatosis (Table 1b). In a multivariable model (Table 2a), pre-HCT hepatic steatosis was not associated with development of grade II-IV acute GvHD (aGvHD) (OR 0.92, 95% CI 0.24–3.57, overall p = 0.90). However, there were three patients who developed acute GvHD of the liver and all had hepatic steatosis (7.69%); none of the patients without pre-HCT hepatic steatosis developed acute GvHD of the liver (p = 0.56). Body composition variables such as weight, height, subcutaneous fat, visceral fat and skeletal muscle areas also did not predict grade II-IV aGvHD (Table 2a). Post transplant cyclophosphamide was protective against grade II-IV aGvHD (OR 0.07, 95% CI 0.01–0.36, overall p <0.001). Unrelated donor raised grade II-IV aGvHD risk (OR 3.57, 95% CI 1.11–11.47, overall p = 0.03).

**Table 2:** a. Predicting aGvHD grade II-IV. Variables in this model were selected from age, race, sex, disease, conditioning, KPS, HCT-CI, and donor type; significant level for entry = 0.1 and significant level for stay = 0.2, other variables are forced in the model (N = 80, event = 53). b. Predicting cGvHD. Variables in this model were selected from age, race, sex, disease, conditioning, KPS, HCT-CI, and donor type; significant level for entry = 0.1 and significant level for stay = 0.2, other variables are forced in the model, i.e. none candidate variable was selected (N = 80, event = 35).

A			
	OR (95% CI)	P-Value	Overall P-Value
Group			
No Hepatic Steatosis	-REF-		0.90
Hepatic Steatosis	0.92 (0.24–3.57)	0.90	
Pre-transplant weight (kg)			
Continuous	1.05 (0.90–1.21)	0.54	0.53
Height (cm)			
Continuous	1.01 (0.92–1.12)	0.78	0.77
Subcutaneous fat at L3			
Continuous	0.99 (0.98–1.01)	0.33	0.32
Visceral fat at L3			
Continuous	1.00 (0.99–1.01)	0.40	0.40
Skeletal muscle at L3			
Continuous	1.01 (0.97–1.04)	0.69	0.69
Race			
White	-REF-		0.08
Other	0.27 (0.06–1.20)	0.09	
Post-transplant cyclophosphamide			
No	-REF-		<0.001
Yes	0.07 (0.01–0.36)	0.001	
Donor Type			
Related	-REF-		0.03
Unrelated	3.57 (1.11–11.47)	0.03	
**B**			
Group			
No Hepatic Steatosis	-REF-		
Hepatic Steatosis	4.19 (1.11–15.75)	0.03	
Pre-transplant weight (kg)			
Continuous	0.99 (0.88–1.10)	0.80	
Height (cm)			
Continuous	0.95 (0.87–1.05)	0.31	
Subcutaneous fat			
Continuous	1.00 (0.99–1.01)	0.53	
Visceral fat			
Continuous	1.00 (0.99–1.01)	0.90	
Skeletal muscle			
Continuous	1.01 (0.98–1.04)	0.45	
Disease			
MPS/MPN/Other	-REF-		
Acute Leukemia	17.17 (1.26–233.70)	0.03	
Lymphomas	1.17 (0.37–3.75)	0.79	

MDS: myelodysplastic syndrome; MPN: myeloproliferative neoplasm

### Chronic GvHD

Chronic GVHD occurred in 29 patients (49.2%) in the hepatic steatosis group and 6 patients (28.6%) in the no hepatic steatosis group (p = 0.13, Table 1b). In a multivariable model (Table 2b), pre-HCT hepatic steatosis was associated with the development of chronic GvHD (cGvHD; OR 4.19, 95% CI [1.11–15.75], overall p = 0.03). Two patients developed chronic GvHD of the liver, both of whom had pre-HCT hepatic steatosis; neither of these patients had acute GvHD of the liver. Body composition variables such as weight, subcutaneous fat, visceral fat, skeletal muscle mass were not associated with cGvHD. A transplant diagnosis of acute leukemia was also associated with development of chronic GvHD (OR 17.17 95% CI [1.26–233.70], p value = 0.03, Table 2b).

### Overall survival and non-relapse mortality

**Fig 2:**
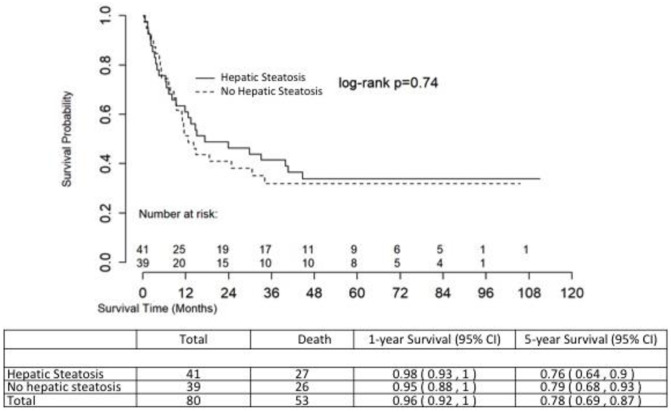
Unadjusted overall survival.

Fig 2 shows the unadjusted overall survival (OS) of the two groups, with no significant difference in OS between those with and without pre-HCT hepatic steatosis (log rank p = 0.74). The multivariable Cox proportional hazard model of overall survival is shown in Table 3. Age at transplant (HR 1.04 (95% CI 1.01–1.06), p value = 0.003) was associated with decreased adjusted overall survival. As expected, aGvHD (HR 2.90 [95% CI 1.47–5.74], p value = 0.002) strongly predicted worse mortality while cGvHD (HR 0.43 [95% CI 0.24–0.79], p = 0.007) seemed to have a protective effect. Those with lower HCT-CI (HR 0.44 [95% CI 0.24–0.81], p value = 0.008) also had improved survival.

**Table 3:** Cox proportional hazard model of adjusted overall survival (with selection from aGvHD, cGvHD, age, race, sex, disease, conditioning, KPS, HCT-CI, and donor type; significant level for entry = 0.1 and significant level for stay = 0.2, other variables are forced in the model) (N = 80, event = 53).

	HR (95% CI)	P-Value
Group		
No Hepatic Steatosis	-REF-	
Hepatic Steatosis	0.57 (0.30–1.11)	0.10
Pre-transplant Weight (kg)		
Continuous	0.95 (0.88–1.02)	0.17
Subcutaneous Fat		
Continuous	1.00 (0.99–1.01)	0.43
Visceral Fat		
Continuous	1.00 (0.99–1.01)	0.30
Skeletal Muscle Mass		
Continuous	1.00 (0.98–1.02)	0.91
Age at Transplant		
Continuous	1.04 (1.01–1.06)	0.003
Acute Graft vs Host Disease grade II-IV		
No	-REF-	
Yes	2.903(1.47–5.74)	0.002
Chronic Graft vs Host Disease		
No	-REF-	
Yes	0.43 (0.24–0.79)	0.007
HCT-CI		
>3	-REF-	
< = 3	0.44 (0.24–0.81)	0.008

HCT-CI: Hematopoietic Cell Transplantation-Comorbidity Index

**Fig 3:**
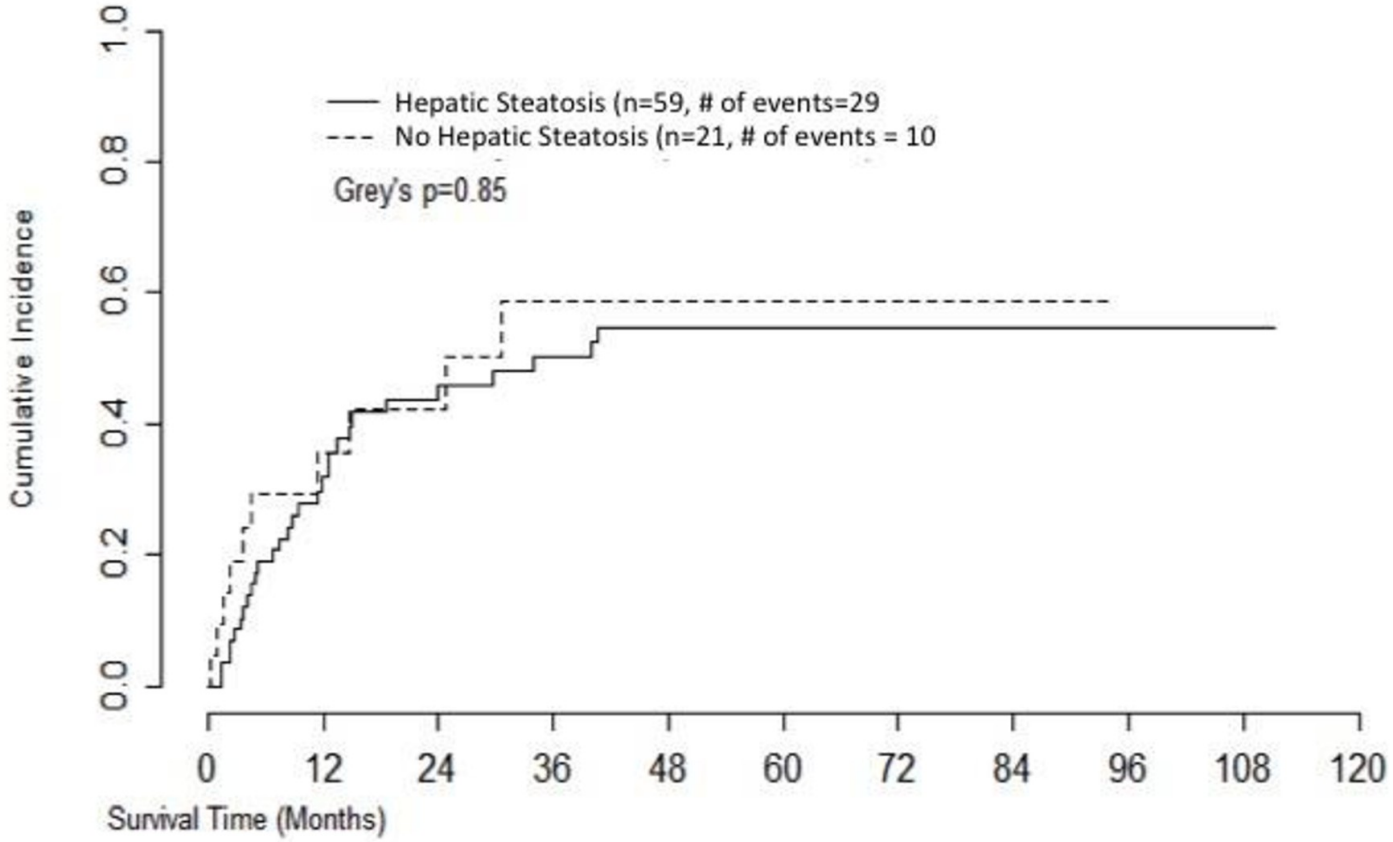
Non-relapse mortality.

Kaplan-Meier curve for non-relapse mortality (NRM) is shown in Fig 3 and mortality was not statistically different between the two cohorts (Grey’s p value = 0.62).

## Discussion

Our study examined the relationship between pre-allo HCT hepatic steatosis and transplant outcomes including GvHD. Hepatic steatosis was not found to be an independent predictor of acute GvHD, however it did strongly predict chronic GvHD in our study population. Furthermore, all patients who developed acute GVHD of the liver and chronic GVHD of the liver were found to have pre-HCT hepatic steatosis. Other factors previously associated with risk of developing GVHD, (including acute leukemia, HLA disparity, conditioning intensity, and post-transplant cyclophosphamide) were confirmed in our multivariable models for acute and/or chronic GVHD, lending validity to the models. While higher age in the group of patients with hepatic steatosis (a relationship previously described in the literature [27]) may have contributed to our findings, the hepatic steatosis group did not have higher overall mortality or non-relapse mortality suggesting any effect of age on chronic GVHD did not impact mortality.

Prior literature has suggested that metabolic syndrome/obesity may be associated with development of aGVHD and poor HCT outcomes [11, 12]. In our sample, we did not find an association between hepatic steatosis and aGvHD. We did find that hepatic steatosis was associated with cGVHD though it did not predict worse OS or NRM. One potential explanation for the link between hepatic steatosis and GVHD is similarities in the inflammatory reaction they are characterized by. Hepatic steatosis is associated with increased levels of several inflammatory cytokines including IL-1 [14] and IL-6 [28]. The association with IL-1 and hepatic steatosis seems to be especially significant as neutralization in mouse models of NASH with IL-1R antagonist anakinra was associated with attenuated liver damage [14]. IL-1 is important in the regulation of inflammation and T-cell differentiation, factors that are central to the development of GVHD [29]. In fact pre-clinical data suggesting the importance of IL-1 in GVHD pathogenesis [30] led to multiple clinical trials of IL-1 receptor antagonists in humans [31–33], though with mixed results. Furthermore, IL-6 is central to the differentiation of naïve T cells away from a regulatory T-cell lineage in favor of the T_H_17 phenotype, facilitating the inflammatory response [29]. The significant role of IL-6 in GVHD development is illustrated by improved GVHD with use of an IL-6 antagonist [34, 35].

Skeletal muscle depletion (sarcopenia, defined in men as BMI < 25 with muscle mass less than 43 cm2/m2 or BMI ≥ 25 with muscle mass < 53 cm2/m2; in women regardless of BMI, 41 cm2/m2) has also been associated with lower non-relapse mortality and overall poorer outcomes in allogeneic stem cell transplant [36], however we did not find an association between skeletal muscle area and HCT outcomes. Our finding that pre-HCT hepatic steatosis, but not other imaging measures of metabolic syndrome/obesity, is associated with chronic GVHD suggests it may be a uniquely useful marker.

Our study does have certain limitations in addition to those characteristic of all retrospective studies such as the potential for uncontrolled confounders. While all patients who developed liver GVHD had pre-HCT imaging findings with hepatic steatosis, our ability to make definitive associations is limited by our small sample size. Furthermore, our institution does not routinely perform pre-allo-HCT CT scans; therefore, we were limited to subjects who had a scan for other purposes, most commonly pre-HCT staging of their disease, and most of these scans were done with intravenous contrast. Moreover, the scans were not specifically calibrated for the identification of hepatic steatosis: Liver and spleen attenuations in contrast enhanced CT scans can be affected by timing of the contrast administration and lower sensitivity may have compromised our ability to find an association between steatosis and HCT outcomes. In the future, quantification of hepatic steatosis on non-contrasted CT scans could be considered, for instance at centers that do routine pre-HCT CT scans [37]. Other imaging modalities such as ultrasound or MRI, or liver biopsy, could be considered, however these are not routinely done and would need to be evaluated in the setting of a dedicated, prospective trial. Furthermore, a larger prospective study incorporating microbiome and inflammatory marker analysis may allow correlation of imaging and clinical findings with measurable alterations in the systemic inflammatory profile to corroborate findings and suggest underlying mechanisms.

This study suggests that hepatic steatosis may be associated with development of chronic GvHD as well as GVHD of the liver; however, it does not predict acute GvHD or overall/non-relapse mortality. Further evaluation with more optimal imaging techniques and/or liver biopsy in an intervention-based prospective analysis may clarify the predictive relationship between pre-allo HCT hepatic steatosis and GvHD.

## Supporting information

S1 Table Comparison of the study cohort and the non-study cohort. (DOCX)

S2 Table Comparison of two cohorts of patients with and without clinically significant acute GvHD, grade II-IV. (DOCX)

S3 Table Comparison of two cohorts of patients with and without chronic GvHD. (DOCX)

S1 Data (CSV)
