# Supplementary material for: Pre-transplant hepatic steatosis (fatty liver) is associated with chronic graft-vs-host disease but not mortality
Source: PLoS One. 2020 Sep 11;15(9):e0238824. doi: 10.1371/journal.pone.0238824 (PMC7485815; doi:10.1371/journal.pone.0238824)
Supplement: S1 Table — (DOCX) [file pone.0238824.s001.docx]

Supplemental Table 1: Comparison of the study cohort and the non-study cohort

|  | **All Patients** | **Study Cohort** | **Non-Study Cohort** |  |
| --- | --- | --- | --- | --- |
|  | **N=692**  **(100%)** | **N=80**  **(11.6%)** | **N=612**  **(88.4%)** | **P-Value** |
| Age at Transplant | | | | |
| Median (IQR) | 52 (40 - 60.5) | 49 (34.5 - 57.5) | 52.5 (42 - 61) | 0.004 |
| Sex | | | | |
| Female | 280 (40.5%) | 31 (38.8%) | 249 (40.7%) | 0.74 |
| Pre-transplant Weight (kg) | | | | |
| Median (IQR) | 83.48 (71.16 - 95.95) | 80.15 (70.75 - 92.5) | 83.9 (71.16 - 96.45) | 0.33 |
| Height (cm) | | | | |
| Median (IQR) | 172.8 (165 - 180.3) | 172.8 (164.5 - 179.05) | 172.85 (165 - 180.3) | 0.40 |
| Disease | | | | |
| Acute Leukemias | 347 (50.1%) | 7 (8.8%) | 340 (55.6%) | <0.001 |
| Lymphomas | 146 (21.1%) | 67 (83.8%) | 79 (12.9%) |  |
| MDS/MPN/Other | 199 (28.8%) | 6 (7.5%) | 193 (31.5%) |  |
| Conditioning Class | | | | |
| Myeloablative | 421 (60.8%) | 31 (38.8%) | 390 (63.7%) | <0.001 |
| Non-myeloablative | 271 (39.2%) | 49 (61.3%) | 222 (36.3%) |  |
| Cell Type | | | | |
| Bone Marrow | 66 (9.5%) | 2 (2.5%) | 64 (10.4%) | 0.01 |
| Cord | 111 (16%) | 7 (8.8%) | 104 (17%) |  |
| Peripheral Blood Progenitor Cells | 515 (74.4%) | 71 (88.8%) | 444 (72.5%) |  |
| Donor Type | | | | |
| Related | 276 (39.9%) | 36 (45%) | 240 (39.2%) | 0.32 |
| Unrelated | 416 (60.1%) | 44 (55%) | 372 (60.8%) |  |
| HLA Match | | | | |
| Matched | 501 (72.4%) | 65 (81.3%) | 436 (71.2%) | 0.06 |
| Unmatched | 191 (27.6%) | 15 (18.8%) | 176 (28.8%) |  |
| KPS | | | | |
| <=80 | 309 (44.7%) | 40 (50%) | 269 (44%) | 0.37 |
| >80 | 338 (48.8%) | 36 (45%) | 302 (49.3%) |  |

MDS: myelodysplastic syndrome; MPN: myeloproliferative neoplasm; HLA: human leukocyte antigens; KPS: Karnofsky Performance Score
