# Supplementary material for: Pre-transplant hepatic steatosis (fatty liver) is associated with chronic graft-vs-host disease but not mortality
Source: PLoS One. 2020 Sep 11;15(9):e0238824. doi: 10.1371/journal.pone.0238824 (PMC7485815; doi:10.1371/journal.pone.0238824)
Supplement: S2 Table — (DOCX) [file pone.0238824.s002.docx]

Supplemental Table 2: comparison of two cohorts of patients with and without clinically significant acute GvHD, grade II-IV

|  | **All Patients** | **aGvHD, 0-I** | **aGvHD, II-IV** |  |
| --- | --- | --- | --- | --- |
|  | **N=80**  **(100%)** | **N=27**  **(33.7%)** | **N=53**  **(66.3%)** | **P-Value** |
| Age at Transplant (years) | | | | |
| Median (IQR) | 49 (34.5 - 57.5) | 52 (36 - 57) | 45 (34 - 58) | 0.81 |
| Sex | | | | |
| Female | 31 (38.8%) | 13 (48.1%) | 18 (34%) | 0.22 |
| Pre-transplant Weight (kg) | | | | |
| Median (IQR) | 80.15 (70.75 - 92.5) | 80.1 (68 - 100.4) | 80.2 (72.7 - 91) | 0.65 |
| Height (cm) | | | | |
| Median (IQR) | 172.8 (164.5 - 179.05) | 172 (163 - 179) | 174 (166 - 179.5) | 0.40 |
| Disease | | | | |
| Acute Leukemias | 6 (7.5%) | 2 (7.4%) | 4 (7.5%) | 0.79 |
| Lymophomas | 54 (67.5%) | 17 (63%) | 37 (69.8%) |  |
| MPS/MPN/Other | 20 (25%) | 8 (29.6%) | 12 (22.6%) |  |
| Conditioning Class | | | | |
| Myeloablative | 31 (38.8%) | 14 (51.9%) | 17 (32.1%) | 0.10 |
| Non-myeloablative | 49 (61.3%) | 13 (48.1%) | 36 (67.9%) |  |
| Cell Type | | | | |
| Bone Marrow | 2 (2.5%) | 2 (7.4%) | 0 (0%) | 0.03 |
| Cord Blood | 7 (8.8%) | 4 (14.8%) | 3 (5.7%) |  |
| Peripheral Blood Progenitor Cells | 71 (88.8%) | 21 (77.8%) | 50 (94.3%) |  |
| Donor Type | | | | |
| Related | 36 (45%) | 17 (63%) | 19 (35.8%) | 0.02 |
| Unrelated | 44 (55%) | 10 (37%) | 34 (64.2%) |  |
| HLA Match | | | | |
| Matched | 65 (81.3%) | 21 (77.8%) | 44 (83%) | 0.56 |
| Unmatched | 15 (18.8%) | 6 (22.2%) | 9 (17%) |  |
| KPS | | | | |
| <=80 | 43 (53.8%) | 13 (48.1%) | 30 (56.6%) | 0.47 |
| >80 | 37 (46.3%) | 14 (51.9%) | 23 (43.4%) |  |
|  | | | | |
| Post-transplant Cyclophosphamide | 16 (20%) | 10 (37%) | 6 (11.3%) | 0.02 |
| Anti-thymocyte Globulin | 9 (11.3%) | 3 (11.1%) | 6 (11.3%) | >0.99 |

MDS: myelodysplastic syndrome; MPN: myeloproliferative neoplasm; HLA: human leukocyte antigens; KPS: Karnofsky Performance Score
