# Supplementary material for: Pre-transplant hepatic steatosis (fatty liver) is associated with chronic graft-vs-host disease but not mortality
Source: PLoS One. 2020 Sep 11;15(9):e0238824. doi: 10.1371/journal.pone.0238824 (PMC7485815; doi:10.1371/journal.pone.0238824)
Supplement: S3 Table — (DOCX) [file pone.0238824.s003.docx]

Supplemental Table 3: comparison of two cohorts of patients with and without chronic GvHD

|  | **All Patients** | **cGvHD 0** | **cGvHD I-IV** |  |
| --- | --- | --- | --- | --- |
|  | **N=80**  **(100%)** | **N=45**  **(56.2%)** | **N=35**  **(43.8%)** | **P-Value** |
| Age at Transplant (years) | | | | |
| Median (IQR) | 49 (34.5 - 57.5) | 52 (34 - 59) | 48 (35 - 56) | 0.43 |
| Sex | | | | |
| Female | 31 (38.8%) | 17 (37.8%) | 14 (40%) | 0.84 |
| Pre-transplant Weight (kg) | | | | |
| Median (IQR) | 80.15 (70.75 - 92.5) | 81.5 (70.2 - 93.4) | 80.1 (72 - 91.4) | 0.83 |
| Height (cm) | | | | |
| Median (IQR) | 172.8 (164.5 - 179.05) | 175 (167 - 180.3) | 170 (163 - 177) | 0.08 |
| Disease | | | | |
| Acute Leukemias | 6 (7.5%) | 1 (2.2%) | 5 (14.3%) | 0.13 |
| Lymophomas | 54 (67.5%) | 32 (71.1%) | 22 (62.9%) |  |
| MPS/MPN/Other | 20 (25%) | 12 (26.7%) | 8 (22.9%) |  |
| Conditioning Class | | | | |
| Myeloablative | 31 (38.8%) | 16 (35.6%) | 15 (42.9%) | 0.64 |
| Non-myeloablative | 49 (61.3%) | 29 (64.4%) | 20 (57.1%) |  |
| Cell Type | | | | |
| Bone Marrow | 2 (2.5%) | 2 (4.4%) | 0 (0%) | 0.62 |
| Cord Blood | 7 (8.8%) | 4 (8.9%) | 3 (8.6%) |  |
| Peripheral Blood Progenitor Cells | 71 (88.8%) | 39 (86.7%) | 32 (91.4%) |  |
| Donor Type | | | | |
| Related | 36 (45%) | 22 (48.9%) | 14 (40%) | 0.43 |
| Unrelated | 44 (55%) | 23 (51.1%) | 21 (60%) |  |
| HLA Match | | | | |
| Matched | 65 (81.3%) | 35 (77.8%) | 30 (85.7%) | 0.40 |
| Unmatched | 15 (18.8%) | 10 (22.2%) | 5 (14.3%) |  |
| KPS | | | | |
| <=80 | 43 (53.8%) | 25 (55.6%) | 18 (51.4%) | 0.71 |
| >80 | 37 (46.3%) | 20 (44.4%) | 17 (48.6%) |  |
|  | | | | |
| Post-transplant Cyclophosphamide | 16 (20%) | 11 (24.4%) | 5 (14.3%) | 0.40 |
| Anti-thymocyte Globulin | 9 (11.3%) | 6 (13.3%) | 3 (8.6%) | 0.72 |

MDS: myelodysplastic syndrome; MPN: myeloproliferative neoplasm; HLA: human leukocyte antigens; KPS: Karnofsky Performance Score
